# Differential expression of interferon-induced protein with tetratricopeptide repeats 3 (*IFIT3*) in Alzheimer's disease and HIV-1 associated neurocognitive disorders

**DOI:** 10.1038/s41598-022-27276-7

**Published:** 2023-02-25

**Authors:** Armando Garces, Bryan Martinez, Roberto De La Garza, Deepa Roy, Kaylie-Anna Vallee, Jerel Adam Fields, David J. Moore, Hansapani Rodrigo, Upal Roy

**Affiliations:** 1 Department of Health and Biomedical Sciences, University of Texas Rio Grande Valley, One West University Blvd, Brownsville, TX 78520 USA; 2 Department of Psychiatry, University of California, San Diego, La Jolla, CA USA; 3 School of Mathematical and Statistical Sciences, University of Texas Rio Grande Valley (UTRGV), Edinburg, TX USA

**Keywords:** Computational biology and bioinformatics, Neuroscience

## Abstract

The United Nations projects that one in every six people will be over the age of 65 by the year 2050. With a rapidly aging population, the risk of Alzheimer's disease (AD) becomes a major concern. AD is a multifactorial disease that involves neurodegeneration in the brain with mild dementia and deficits in memory and other cognitive domains. Additionally, it has been established that individuals with Human Immunodeficiency Virus-1 (HIV-1) experience a 5 to 10-year accelerated aging and an increased risk of developing HIV-associated neurocognitive disorders (HAND). Despite a significant amount of clinical evidence pointing towards a potential overlap between neuropathogenic processes in HAND and AD, the underlying epigenetic link between these two diseases is mostly unknown. This study is focused on identifying differentially expressed genes observed in both AD and HAND using linear regression models and a more robust significance analysis of microarray. The results established that the dysregulated type 1 and 2 interferon pathways observed in both AD and HAND contribute to the similar pathologies of these diseases within the brain. The current study identifies the important roles of interferon pathways in AD and HAND, a relationship that may be useful for earlier detection in the future.

## Introduction

Alzheimer's disease (AD) has become a growing concern among people over the age of 65 as advances in medical science contribute to increased longevity among persons with Human Immunodeficiency Virus -1 (HIV-1). The multifactorial pathology of AD that affects the central nervous system (CNS) has been observed in other diseases, including HIV-1 infection^1–3^. Before effective combination antiretroviral therapy (cART), HIV-1 infection could lead to HIV-associated dementia (HAD) if left untreated. Due to the advancements in cART, this form of dementia is extremely rare, and milder forms of HIV-associated neurocognitive disorders (HAND) are more common among treated patients^4^. There is significant clinical evidence indicating a potential link between the neurodegeneration developed during HAND and AD. However, the relationship between HAND and AD is still not well characterized^4–6^. Therefore, the current study investigated an in situ genome-wide screening of transcription regulators in brain tissue, identifying genes that can be used as biomarkers for early detection.

According to the Alzheimer's Association, AD is the most common form of dementia in the United States. In 2020, approximately 5.8 million people over the age of 65 were living with AD in the United States^7^. AD is a complex neurodegenerative disorder that is associated with the accumulation of amyloid-beta and neurofibrillary tangles in regions of the brain^8^. Late detection of AD is the most common, accounting for 96–99% of all cases. Early stages of AD can progress without displaying any symptoms in patients^8,9^. Currently, there is only one FDA-approved intervention to slow the progression of neurodegeneration in AD (i.e., aducanumab), and it is not without controversy. Therefore, early detection and intervention are crucial to halt AD progression^10^.

According to the United Nations AIDS (UNAIDS) organization, in 2021, 38.4 million people were living with HIV-1 worldwide, and 1.2 million of these patients were in the United States^6,11,12^. Even though cART has successfully suppressed viral loads, HAND affects up to 50% of HIV-1 patients treated with cART^5^. This may be due to the brain's role as a reservoir for HIV-1, where the virus enters the brain early during infection and has been detected in the white matter of post-mortem brain tissues^13^. Many studies have focused on investigating brain tissue samples to identify the key pathophysiological features of patients with different severity levels of HAND^13^. Previous studies comparing cortical and subcortical regions have shown that studying multiple brain regions of people with HIV-1 (PWH) will help predict neuropsychological impairment^14^. Interferon response genes (IFRG) are related to the cell functions in the immune system and autoimmunity and have been found differentially expressed in the brains of persons with HAND^15–17^. Early in HIV-1 infection, the virus enters the brain and begins replicating within the microglia and perivascular macrophages, eventually causing neuronal damage that promotes the onset of the symptoms associated with HAND^18^. A recent study has found that there may be similar early pathophysiology in HAND and AD^5^. The compounding effects of HIV-1 and its influence on aging urge further investigation to determine the pathology of AD and HAND among PWH to improve prognosis and treatment outcomes^6^. Therefore, developing a set of biomarkers that can differentiate and detect AD and HAND in aging individuals with HIV-1 is crucial^19^.

The accumulation of extracellular amyloid-beta protein and intracellular hyperphosphorylated tau protein in various brain regions have been the two main hallmarks of AD since they were identified more than 100 years ago^20^. In addition, comparative studies indicate that amyloid plaques were observed in HAND patients, suggesting that HAND and AD may be interrelated^21,22^. Similarly, studies demonstrate that cART treatment is associated with increased amyloid-beta levels in the cerebral spinal fluid (CSF) of people with asymptomatic neurocognitive impairment (ANI) and decreased levels in individuals with HIV-associated dementia (HAD)^23^. While these data suggest that amyloid-beta clearance varies between HAND groups, the relationship between amyloid-beta levels in CSF and cognitive impairment deserves further investigation^14^. Triggering receptor expressed on myeloid (TREM) 2 has been associated with AD and has various functions, including neuroinflammation regulation and the clearance of amyloid-beta^24^. Brain protein levels and cellular localization of TREM2 were also found altered in PWH with HAND, suggesting a common pathogenic mechanism in the two neurodegenerative diseases^15^. The interaction of amyloid-beta in people infected with HIV-1 can disrupt various processes that may lead to AD, including the development of neurodegeneration.

This study identifies a set of genes that are significantly involved in AD and HAND pathologies using linear regression models and significance analysis of microarray (SAM). Datasets accessed through an NCBI database containing total RNA data were analyzed independently to healthy controls to identify differentially expressed genes (DEGs). Independent results were compared to determine common DEGs involved in key pathways that lead to neurodegeneration. This information will contribute to the identification of potential biomarkers that can detect the early neurodegeneration common in the aging population with AD and PWH. Overall, the present study implicates the dysregulation of the type 1 interferon pathway in human post-mortem brains may directly or indirectly regulate AD pathology in PWH.

## Materials and methods

### Subjects

This study investigated three transcriptome profiles containing either HAND or AD-derived patient data. The mRNA-based datasets were obtained from the Gene Expression Omnibus (GEO) database maintained by the National Center for Biotechnology Information^25^. The available specific details of the data sets can be found in Table 1. The mean age at death for subjects in dataset GSE35864 with HAD was 43.7 (SD: 9.759) years, and for those with HIV-1, it was 49.5 (SD: 8.334) years, and for the control group, it was 50.0 (SD: 10.373 ) years. Dataset GSE35864 contained 6 healthy controls, 6 cognitively normal people with HIV-1, and 7 people with HAD. The data from GSE35864 was derived from the frontal cortex, basal ganglia, and white matter. At least one comprehensive neurocognitive evaluation was assessed 6 months before death; the test measured attention, working memory, language, motor function skills, and executive functions. In the GSE35864 dataset, the control group had 5 males and 1 female, for the HAND there were 7 males and no females. In this study, there were 8 White, 3 Hispanic, 1 Black, and 1 Mixed Asian participant involved. The ages of subjects in the dataset GSE28160, which contains HAND data, ranged from 21 to 64 years with a mean age of 46 (SD: 11.9159). The data from GSE28160 pertained to 6 healthy controls, 7 cART-treated people with HAND (treated HAND), and 8 untreated people with HAND (untreated HAND).In the GSE28160 dataset, the control group had 3 males and 3 females. The untreated HAND group had 7 males with 1 female. The cART-treated HAND group had 6 males and 1 female. The dataset contained data from 8 White, 7 Hispanic, and 6 Black participants. All of the data from dataset GSE28160 was derived from the white matter of post-mortem brains. HAND was determined using neuropsychological evaluation and post-mortem neuropathology.

**Table 1:** Gene expression data summary.

Data set ID	Pathology (HAND/AD)	Database (platform)	Brain regions analyzed	Different subject types
GSE 35864	HAND	GEO (GPL 570)	FC, WM, BG	6 controls, 6 HIV-1 infected subjects with none or slight neurocognitive impairment, 7 subjects with HIV-Associated dementia (HAD)
GSE 28160	HAND	GEO (GPL570)	WM	7 treated HAND subjects, 8 untreated HAND subjects, 6 controls
GSE 84422	AD	*GEO (GPL96) *GEO (GPL97) GEO (GPL570)	*FC, *BG, nucleus accumbens/amygdala	*32 subjects with AD, 19 controls; 34 subjects with AD, 19 controls

FC: Frontal Cortex, WM: White Matter, BG: Basal Ganglia.

The asterisks are used to signify that both GPL 96 and GPL97 contain data from 32 subjects with AD and 19 controls.

The AD dataset was found using accession number GSE84422 and contains three platforms described in this research that were categorized based on the Affymetrix chip used to collect data. The age of subjects in the dataset GPL 570 ranged from 62 to 102 years, with a mean age of 87 (SD: 10.291). The AD data contained in this dataset is derived from the nucleus accumbens and amygdala. In GPL 570, there were 42 female and 9 male patients. Although the patient's list was diverse, it was mainly White with 36 patients, 11 Black, 2 Hispanic, and 1 Asian. The ages of AD patients in two GEO platforms (GPL), GPL96 and GPL 97, ranged from 60 to 100 years with a mean age of 86 (SD: 10.523). The data contained in GPL 96 and GPL97 were averaged based on the original publication^26^. Both GPL 96 and GPL97 contained 48 White, 12 Black, 2 Hispanic, and 1 Asian patient, with 41 females and 22 males. These datasets contained data from the frontal cortex and basal ganglia from post-mortem brains. However, unlike the HAND dataset, the AD dataset does not have white matter. Gene expression data were normalized using the Guanine Cytosine Robust Multi-Array Analysis (GCRMA)^27^. GCRMA adjusted for background intensities in Affymetrix array data, including optical noise and non-specific binding^28^.

### Principal component and linear model analyses

Principal component analysis was used to visualize subjects' gene profiles in low dimensional space and investigate the presence of any clustering effect among subjects within the same disease group in each data set. Further analyses were performed with Linear models and Significance Analysis of Microarrays (SAM). These approaches helped identify differentially expressed genes with non-normal distributions and whether the variability of these expression values differs between genes. Filtered log2 gene expression data was utilized to fit linear models for each brain region consisting of the datasets GSE36864. The basal ganglia, white matter, and frontal cortex regions were analyzed. For GSE28160, only the white matter was analyzed, and GSE84422 contained the amygdala and nucleus accumbens, basal ganglia, and frontal cortex regions. This method used weighted least squares with empirical Bayes moderation of the standard errors^29^. The limma Bioconductor package was used to manipulate the data to generate a list of differentially expressed genes.

### Significance Analysis of Microarrays (SAM)

SAM is a popular nonparametric method introduced by Tusher et al. that can be used to identify DEGs among different subject groups^30^. DEGs can be identified after controlling for false discovery rates in large and small samples^31^. SAM helps to identify expression patterns that have minor, yet significant differences between the control and other subject groups. This robust test has the advantage over the regular t-test as it applies even when the samples are normally distributed. The "samr" package in R (version 3.6.1) was used to perform the SAM analysis and report the SAM score (*d*), fold change, and the q-value of each gene. The q-value of a gene represents the false discovery rate of the gene list and includes that gene along with all other more significant genes^31^. It is similar to the well-known "*p* value," except it is adjusted so that many genes can be analyzed to determine the significance of a specific observation. The q-value measures how significant the gene is: as $d>0$ increases, the corresponding q-value decreases. DEGs with at least a two-fold change were identified with a 10% false discovery rate in all SAM analyses. All comparisons were made independently from each using two unpaired method analyses, and then the results were used to identify common DEGs. The comparisons were made using normalized data corresponding to AD vs. control, untreated HAND vs. control, cART-treated HAND vs. control, and HAD vs. control. There were only two comparisons using cognitively normal people with HIV-1, and they were HIV-1 vs. control and HAD vs. HIV-1.

### Gene ontology and pathway analyses

Gene Ontology and pathway analyses help identify the common DEGs' biological, molecular, and cellular functions among the AD and HAND subjects. In this regard, the genes with a q-value of zero from the SAM analysis were used to compose the list of common genes. The list of common genes was analyzed using the official Affymetrix gene symbols using the "Functional Annotation Tool" found on DAVID^32^. This analysis provided information on pathways or processes that are affected in both pathologies, allowing for further exploration of the function of specific genes.

## Results

### Statistical significance of datasets using linear models and principal component analysis

Table S1 (supplementary materials) has details related to all comparisons that have been made among six subject groups (HAD vs. control, cognitively normal HIV-1 vs. control, HAD vs. cognitively normal HIV-1, HAND (cART untreated) vs. control, cART treated normal HIV-1 vs. control and AD vs. control) across all three brain sectors that were identified using linear models. The untreated HAND data and treated HAND data were collected from the white matter. The AD data was collected from the basal ganglia, frontal cortex, nucleus accumbens, and amygdala brain regions. For the comparisons of AD vs. control for the nucleus accumbens and amygdala, basal ganglia, and frontal cortex regions, and the white matter for both HAND (cART untreated) vs. control and cART treated HAND vs. control. The upregulated genes in Table S1 are represented by a log-fold value higher than "1" and down-regulated genes are represented by a log-fold value less than "1".For the results of the comparison of HAD vs. HIV-1 and HIV-1 vs. control, the results in Table S1 are denoted by "1" down-regulated genes are "−1" and genes with no difference are "0". All linear model comparisons were made with a healthy control within each dataset, and only the genes with significant *p* values were considered for further analysis.

**Figure 1:**
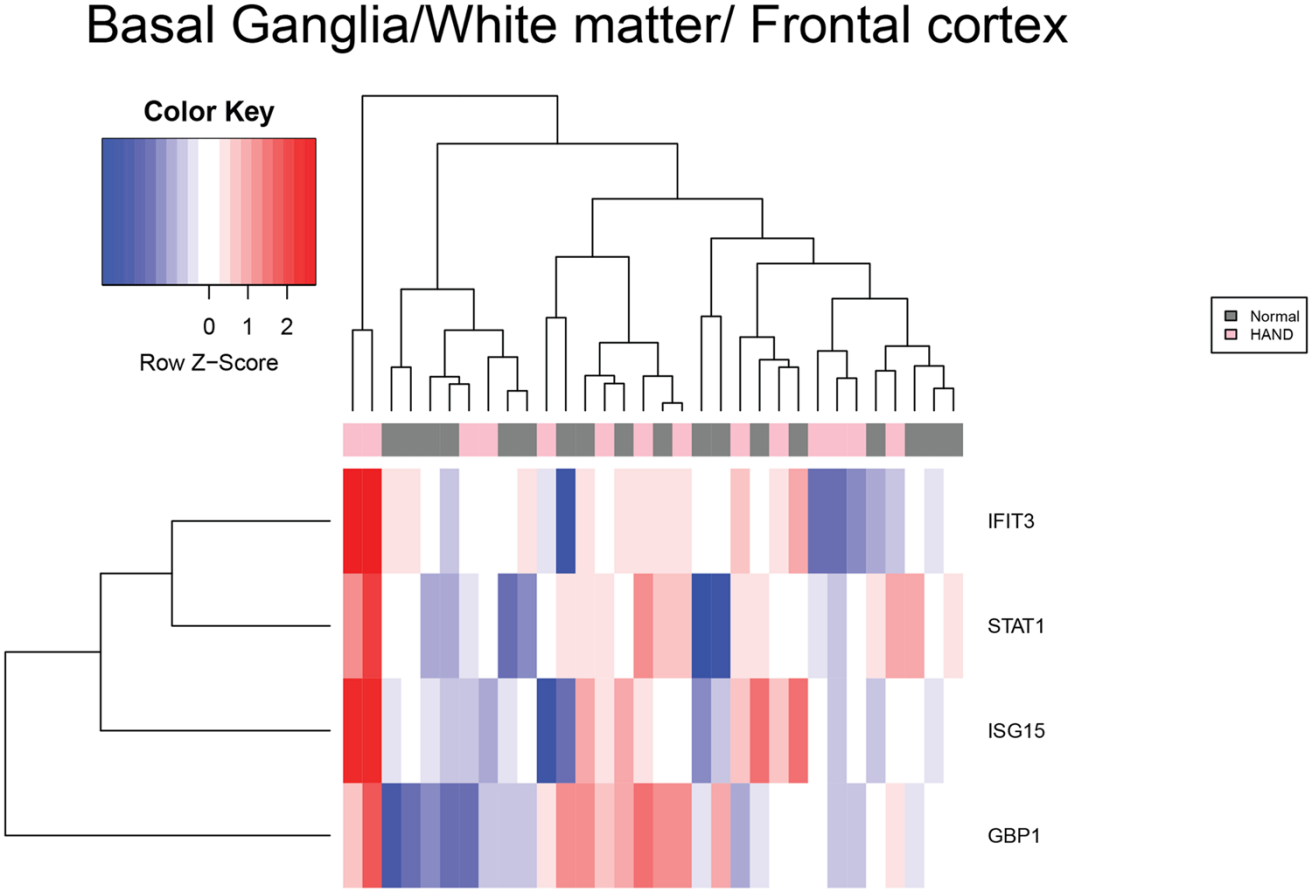
Heatmap of mRNA expression in HAND patients. This figure represents the expression value of mRNA from HAND and controls patients’ brains in GSE35864. The data were normalized using the GCRMA Bioconductor package. The mRNA expression of *IFIT3*, *STAT1, ISG15,* and *GBP1* was chosen by comparing independent SAM results and identifying common markers that were shared between pathologies. The color key represents the Z score the blue represents underexpression and red represents overexpression. These mRNA transcripts were found to be under-expressed in patients with HAD compared to HIV-1 patients.

**Figure 2:**
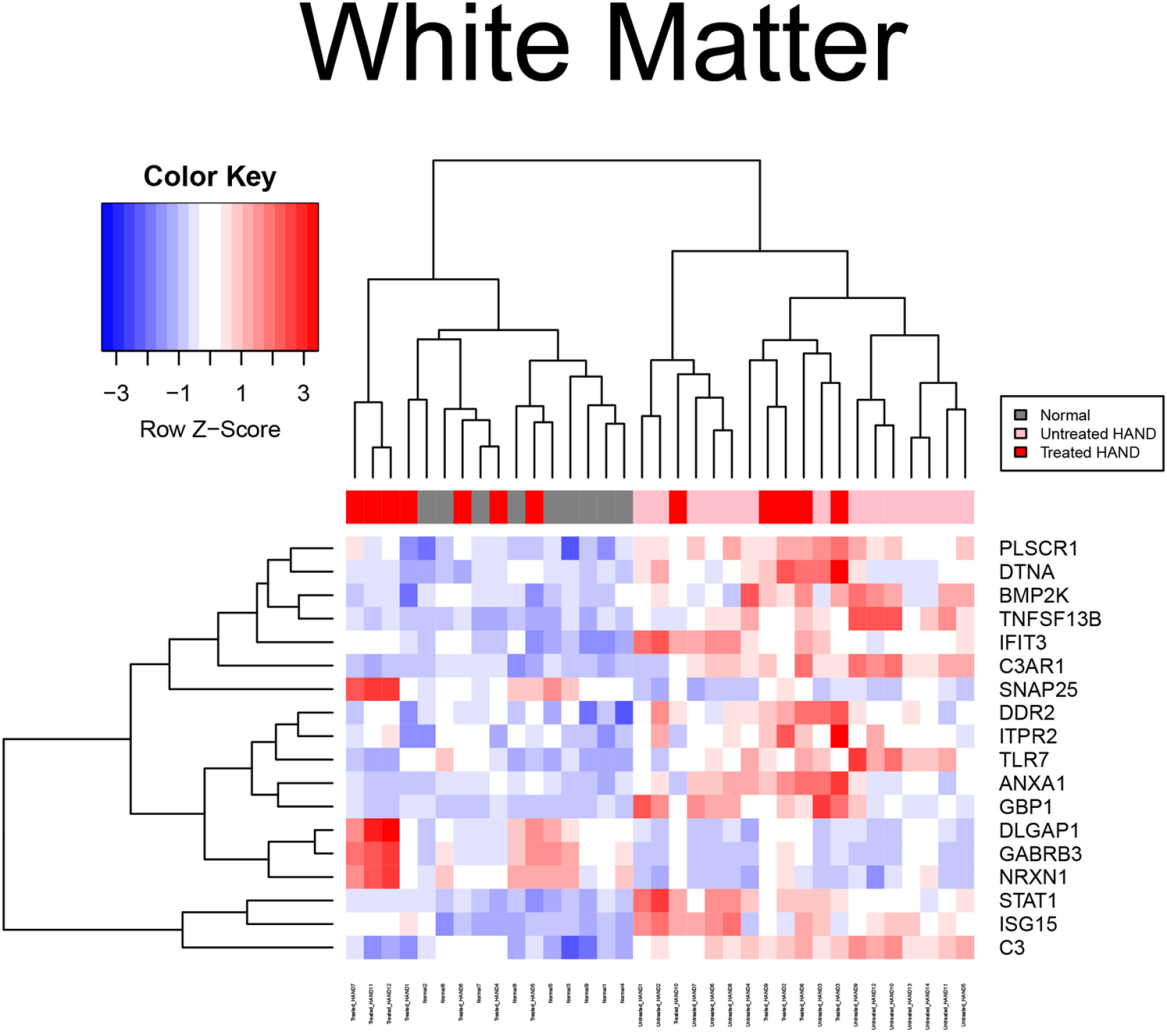
Heatmap of mRNA expression in the white matter of HAND patients. This figure represents the expression value of mRNA from the white matter of untreated HAND and control patients’ brains in GSE28160. The data were normalized using the GCRMA Bioconductor package. The mRNA expression of *PLSCR1, DTNA, BMP2K, IFIT3, DDR2, ITRP2, TLR7, ANXA1, GBP1, SNAP25, NRXN1, DLGAP1, GABRB3, STAT1, ISG15, C3AR1, and C3* was chosen by comparing independent SAM results and identifying common markers that were shared between pathologies. The color key represents the Z score, the blue represents underexpression and the red represents overexpression. The larger sample size makes it easier to see trends compared to the other HAND dataset. We can observe overexpression of mRNA transcripts from untreated HAND including *PLSCR1, DTNA, BMP2K, IFIT3, DDR2, ITRP2, TLR7, ANXA1, GBP1, STAT1, ISG15, C3AR1, and C3*. The mRNA expression of *SNAP25*, *NRXN1*, *DLGAP1*, and *GABRB3* was under-expressed compared to controls.

As shown in Fig. 1, there were no up or down-regulated genes among HAND patients compared to cognitively normal people with HIV-1 in basal ganglia. The z-score indicates the expression levels, and the chart colors highlight whether a gene was over or under-expressed. Figure 2 provides a visual representation of the difference in expression observed between cART-treated and untreated HAND and control subjects after normalization using GCRMA. The cART-treated HAND patient data did not show any significant change in gene expression and hence, were not used for any other comparisons. Nevertheless, the untreated HAND patient data generated a list of 480 DEGs; some of the upregulated genes were observed, including *ISG15, IFIT3, STAT1, TNFSF13B, and GBP1, C3*. Some genes that were upregulated in untreated HAND compared to controls include *STAT1*, *ISG15*, and *C3AR1*.

**Figure 3:**
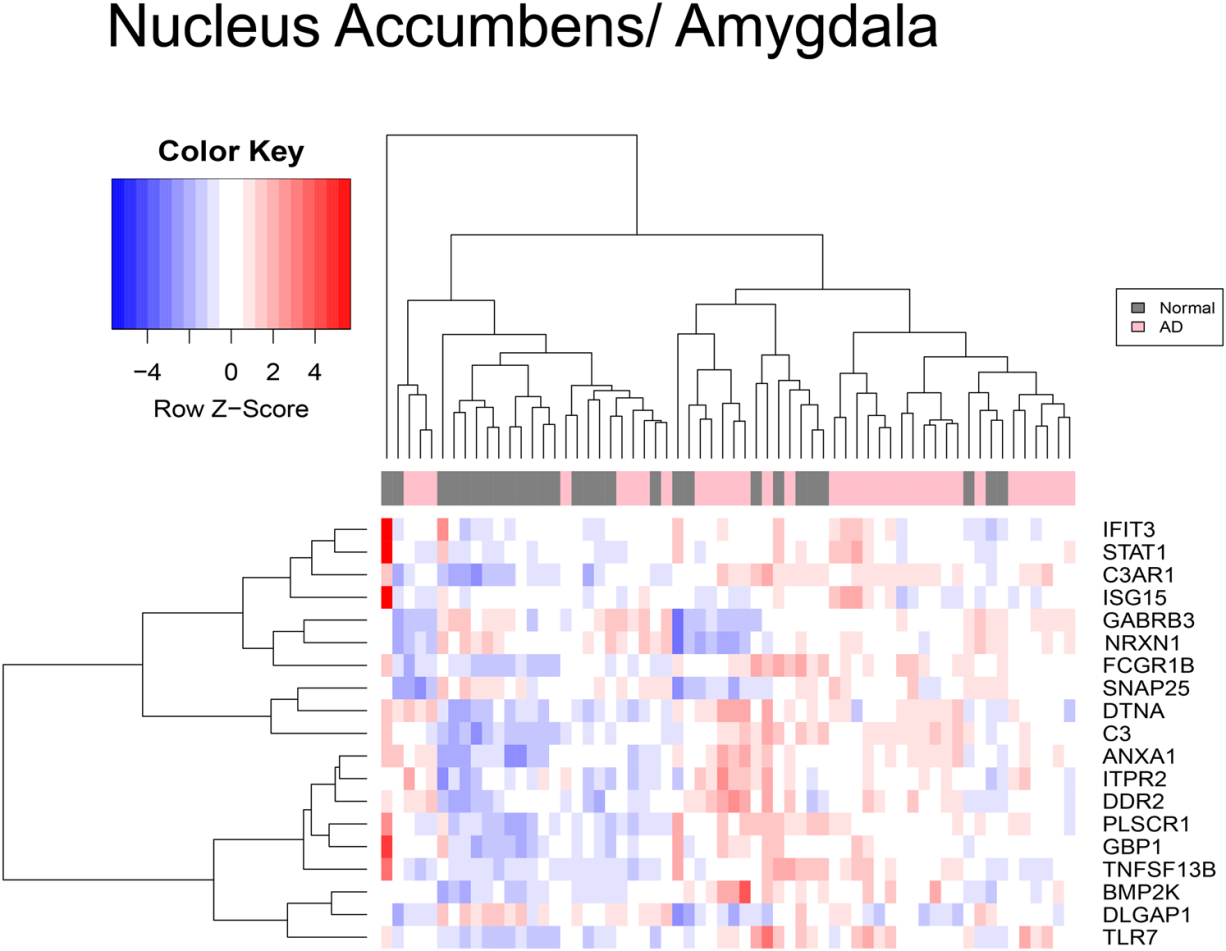
Heatmap of mRNA expression in nucleus accumbens and amygdala AD patients. This figure represents the expression value of mRNA from the Nucleus accumbens and Amygdala of AD and controls patients’ brains in GSE84422. The data were normalized using the GCRMA Bioconductor package. The mRNA expression of *PLSCR1, DTNA, BMP2K, IFIT3, DDR2, ITRP2, TLR7, ANXA1, GBP1, SNAP25, NRXN1, DLGAP1, GABRB3, STAT1, ISG15, C3AR1, and C3* were chosen by comparing independent SAM results and identifying common markers that were shared between pathologies. The color key represents the Z score the blue represents underexpression and red represents overexpression. SAM results demonstrate that mRNA expression of *DTNA* was overexpressed while transcripts *ANXA1, DDR2, ITRP2, BMP2K, C3, C3AR1, and TLR7* were under-expressed.

**Figure 4:**
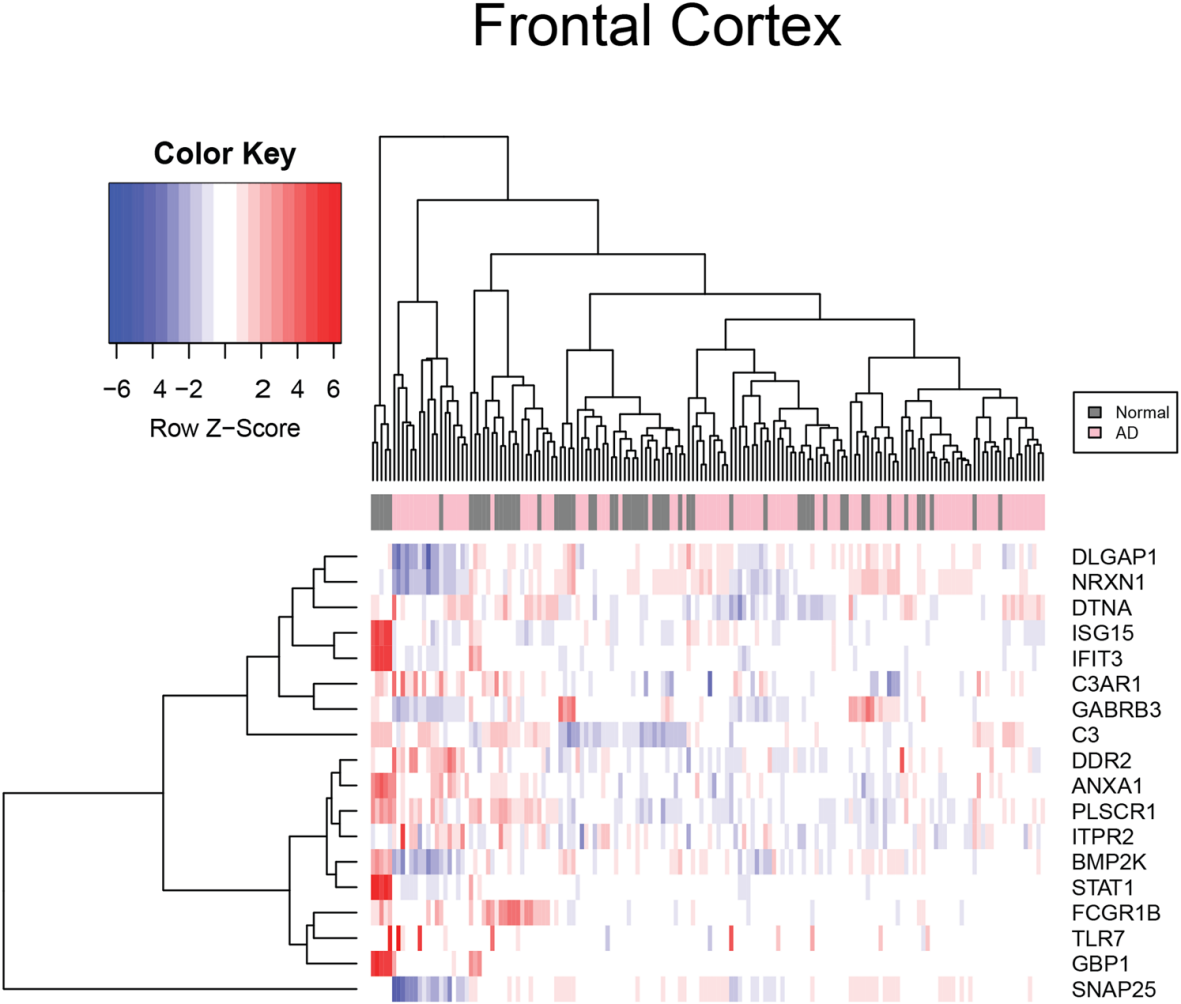
Heatmap of mRNA expression in the frontal cortex AD patients. This figure represents the expression value of mRNA from the frontal cortex regions of AD and control patients’ brains in GSE84422. The data were normalized using the GCRMA Bioconductor package. The mRNA expression of *PLSCR1, DTNA, BMP2K, IFIT3, DDR2, ITRP2, TLR7, ANXA1, GBP1, SNAP25, NRXN1, DLGAP1, GABRB3, STAT1, ISG15, C3AR1, and C3* were chosen by comparing independent SAM results and identifying common markers that were shared between pathologies. The color key represents the Z score the blue represents underexpression and red represents overexpression. SAM results demonstrate the overexpression of *transcripts ISG15, GBP1, IFIT3, DLGAP1 FCGR1B, NRXN1, SNAP25, and GABRB3*.

The data contained in the three platforms that make up the AD dataset were analyzed independently from the HIV-1 dataset as HIV-1 and AD are compared directly for the first time. Figure 3 represents data from the AD dataset's nucleus accumbens and amygdala brain regions. The AD patient data in the platform GPL570 contained 2,985 significant genes. The important transcripts that were downregulated in AD data compared to control in GPL570 were *C3, C3AR1, DDR2, TLR7,* and *ANXA1.* In Fig. 4, the frontal region data contained in GPL 96 had 1,379 DEGs, consisting of *GBP1*, *IFIT3, ISG15, STAT1,* and *DIRAS*. Platform GPL97 contained 575 genes and consisted of *IFIT3*, *GBP1*, *GABRB3*, and *DCLK1*.

**Figure 5:**
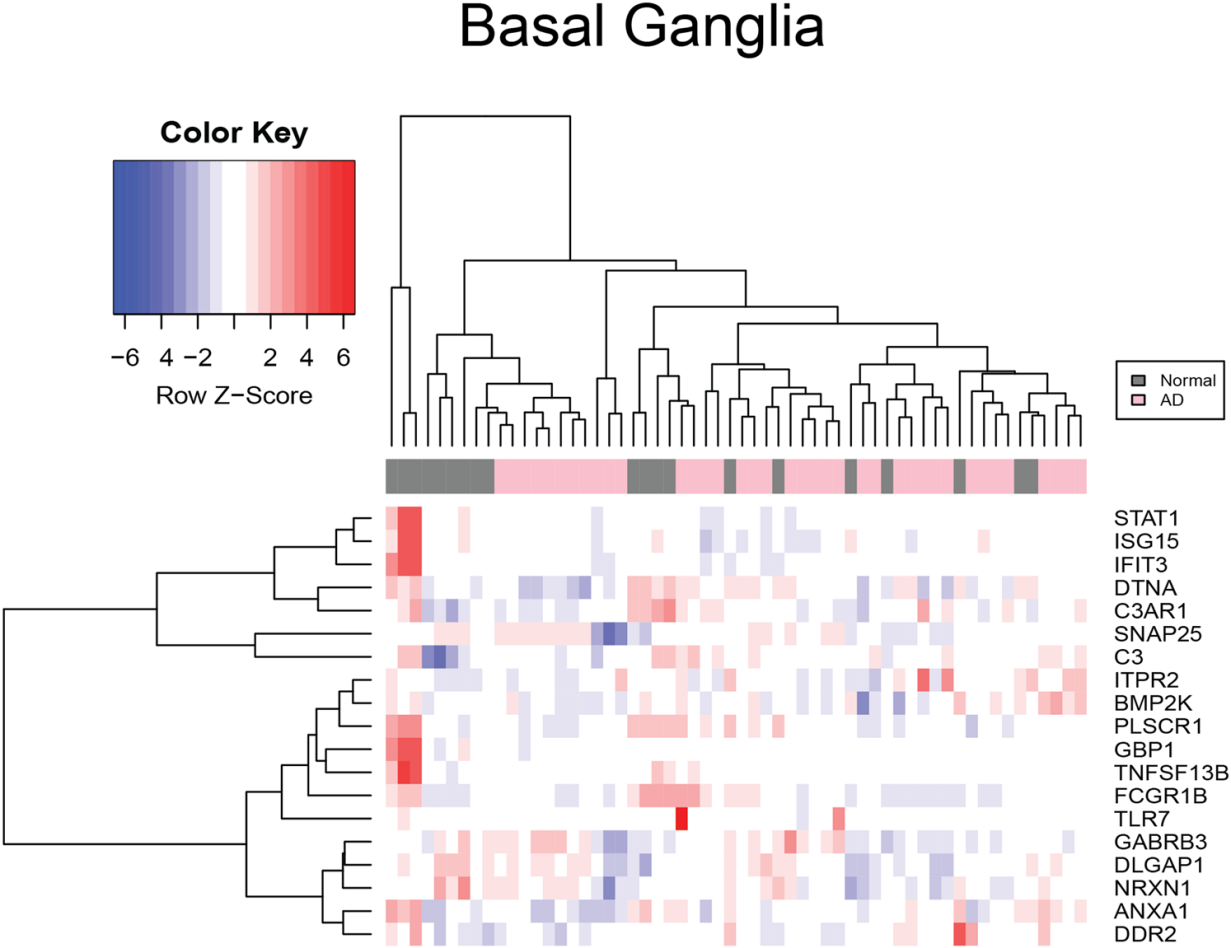
Heatmap of mRNA expression in the basal ganglia of AD patients. This figure represents the expression value of mRNA from the basal ganglia regions of AD and control patients’ brains in GSE84422. The data were normalized using the GCRMA Bioconductor package. The mRNA expression of *PLSCR1, DTNA, BMP2K, IFIT3, DDR2, ITRP2, TLR7, ANXA1, GBP1, SNAP25, NRXN1, DLGAP1, GABRB3, STAT1, ISG15, C3AR1, and C3* were chosen by comparing independent SAM results and identifying common markers that were shared between pathologies. The color key represents the Z score the blue represents underexpression and red represents overexpression. Based on SAM results *ISG15, GBP1, STAT1, PLSCR1, and DTNA* were overexpressed in the basal ganglia of AD subject brains.

Interestingly, IFIT3 appeared to be downregulated in both analyses (platform used). Neither of the basal ganglia regions in GPL96 and GPL97 genes showed significant DEGs when compared to AD vs. controls. Figure 5 represents a heatmap showing the expression levels of the basal ganglia after normalization. There was no significant change in the expression of *STAT1, ISG15*, and *IFIT3* in basal ganglia compared to the frontal cortex when compared to AD vs. control.

**Figure 6:**
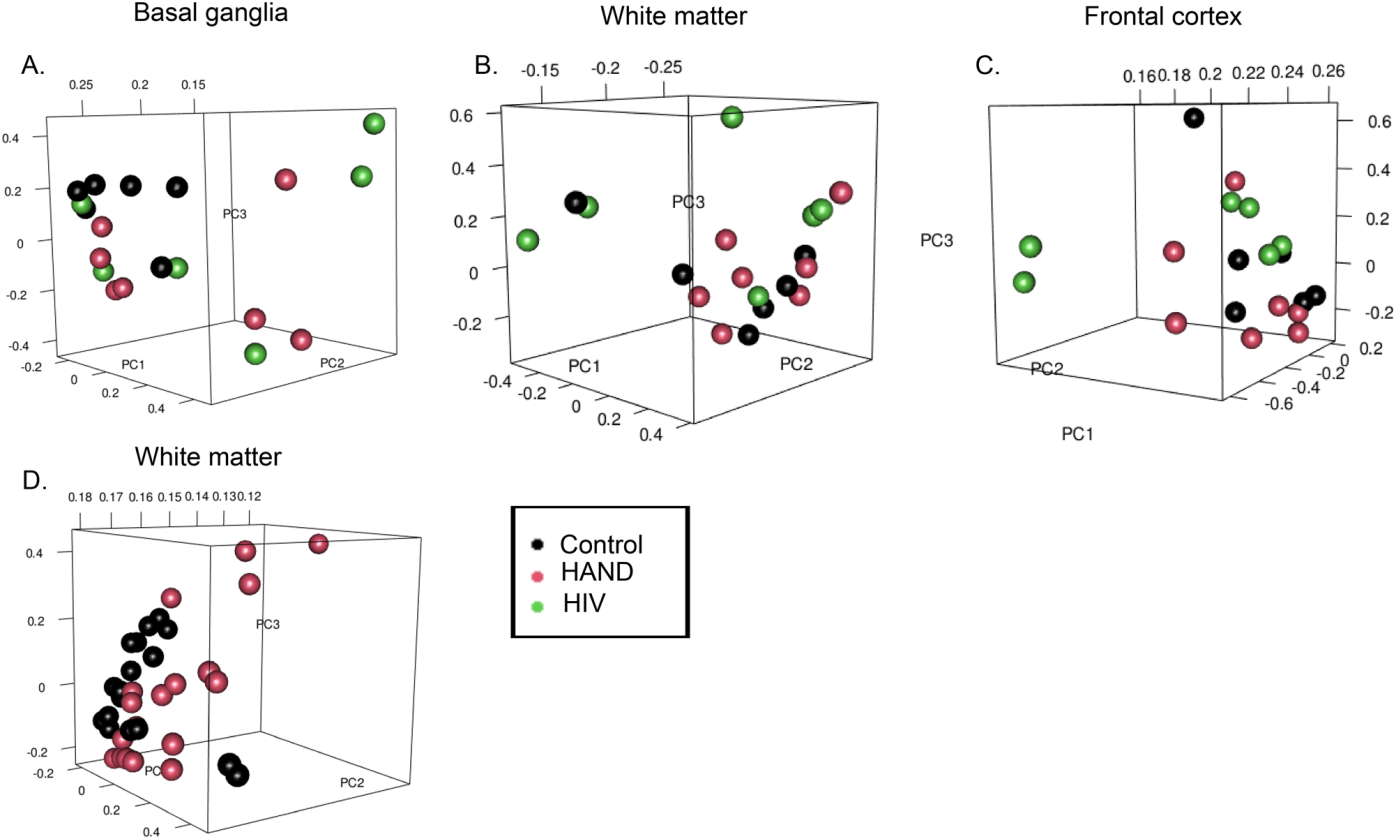
Principal component analysis on HAND patients data. Principal Component Analysis of the controls and other 3 HIV-1 patient groups within the three brain regions, basal ganglia, frontal cortex, and white matter. (**A**–**C**) showed no clear groupings among the different subject groups from data derived from GSE35864. (**D**) represents untreated HAND data from GSE28160.

**Figure 7:**
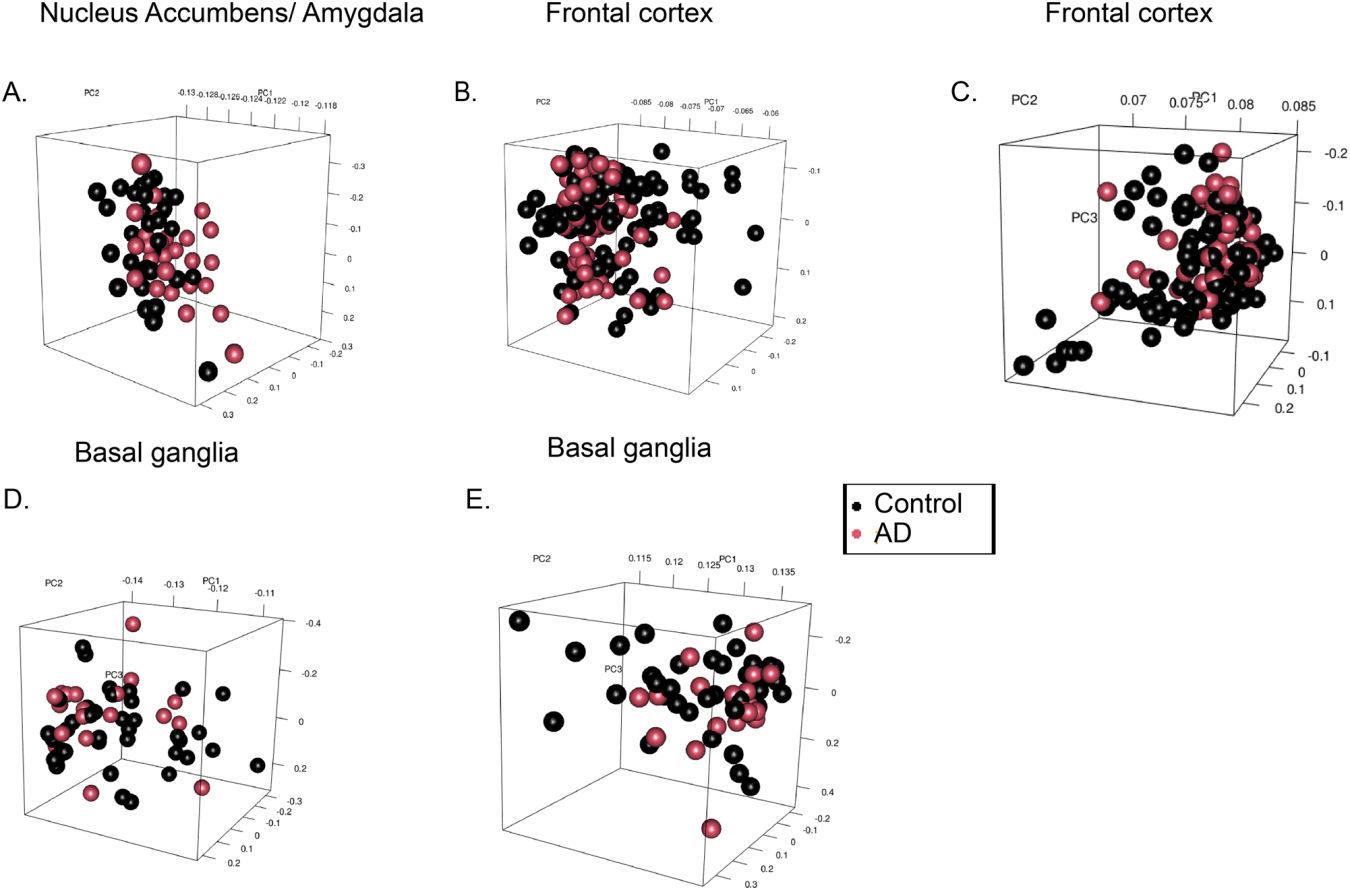
Principal component analysis on AD patients data. Principal Component Analysis of the controls and the three platforms that make up dataset GSE84422. Details of the platforms and patient data can be found in table 1. (**A**) Represents the data derived from the nucleus accumbens and amygdala. This shows some clustering in the negative direction of the PC1 axis in which both the control and AD subject group seem to cluster together within the same area on the axis. AD patients data, however, spread out more and some subject data can be seen on the positive end of the PC1 axis meaning there is more variability in the subject data. (**B**, **C**) represents the frontal cortex data both plots represent clusters of data on opposite ends of the PC1 axis but both contain subject data that is clustered together and show some variability among control groups on the PC2 axis. Figures 7D,E represents the basal ganglia subject data which displays more variability in Fig. 7D with no clear clusters. However, (**E**) depicts clusters on the positive end of the PC1 plot and towards the negative end of the PC2 axis.

Due to a large number of white participants in the AD dataset an additional comparison of AD vs. control using only white participant data was analyzed. For the nucleus accumbens/amygdala data contained in platform GPL570, there were 5343 significant DEGs, and a significant downregulation in *C3*, *C3AR1*, and *DDR2*. This is consistent with the original analysis consisting of all the participants. In further analysis, the gene TLR7 was not significantly downregulated as per the adjusted *p* value. The frontal cortex data found in GPL 96 had 1547 significant DE. The frontal cortex data found in GPL 97 resulted in 798 significant DEGs.Based on the adjusted *p* value there was no significant downregulation of *IFIT3*, *ISG15*, *STAT1*, *GBP1*, *DCLK1*, and *GABRB3*. In the basal ganglia data found on GPL96, there were 6 significant DEGs and in GPL97 there were only 1. This is similar to the results gathered from the original analysis composed of all the participants in which there were no significant DEGs in the basal ganglia. The PCA plots (Figs. 6 and 7) compared the individual expression of each subject within each dataset. An individual sphere represents each subject, and if the subject data was similar then it was observed to be clustered with proximity. If there was more variation between the subject data, the spheres were observed to be further apart. The sample data from distinct brain regions correspond to the top three principal components: black represents the control, HAND is red, and an HIV-1 infected individual without HAND is green. There were no clear groupings among the three subject groups (Fig. 6A–C). In the cART untreated HAND group (Fig. 6D), clustering was found among the control subjects and HIV-1 subjects. However, a few outliers whose expression varied from the rest of the group are between 0 and 0.2 on the PC2 axis, above 0.2 on PC3, and between 0.15 and 0.12 on PC1.

In Fig. 7, the AD subject data is represented in red, and the control subjects are represented in black. The nucleus accumbens and amygdala (Fig. 7A) showed a significant amount of clustering in the negative direction of the PC1 axis, where both control and AD groups seemed to cluster together within the same area on the axis. AD data, however, spread out more, and some were seen on the positive end of the PC1 axis, indicating more variability of the subject data. Both frontal cortex plots (Fig. 7B,C) showed data clusters on opposite ends of the PC1 axis. However, they both contained subject data clustered together and showed some variability among control groups along the PC2 axis. Figure 7D and E represent the basal ganglia subject data from platforms GPL96 and GPL97. More variability was observed in the expression of genes among AD basal ganglia samples seen in Fig. 7D. However, some clusters of the control were seen spread throughout the PC1 axis. AD clusters were observed between −0.14 and −0.13 on the PC1 axis. Within the basal ganglia, GPL97 showed clusters on the positive end of the PC1 axis towards 0.135 and between 0 and −0.2 on the negative end of the PC2 axis (Fig. 7E). These clusters demonstrated similar expressions in AD subjects used for the analysis. The difference seen in RNA expression may be due to the different chips used to analyze the patient samples; this is because of the range of RNA the Affymetrix was probed with.

### Identification of DE genes through SAM analysis

**Figure 8:**
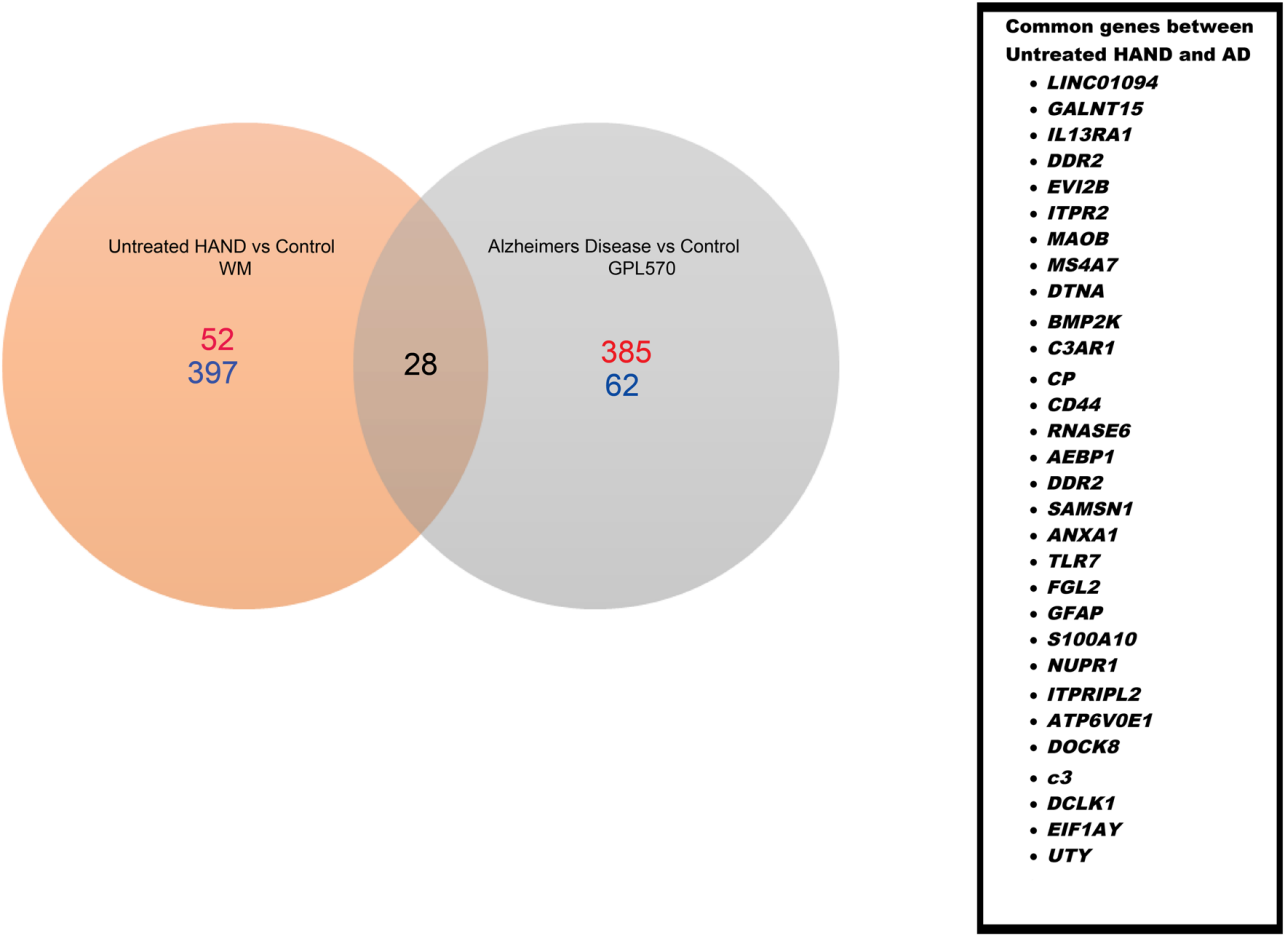
SAM results in comparison of untreated HAND and AD patients (nucleus accumbens/amygdala). This Venn diagram represents the SAM results that were considered significant (q-value =  < .05). Both SAM analyses were done independently and then compared. The white matter of untreated HAND patients had a total of 397 upregulated genes (blue) and 52 downregulated genes (red). The nucleus accumbens and amygdala of AD patients resulted in 62 upregulated genes (blue) and 385 downregulated genes (red).

**Figure 9:**
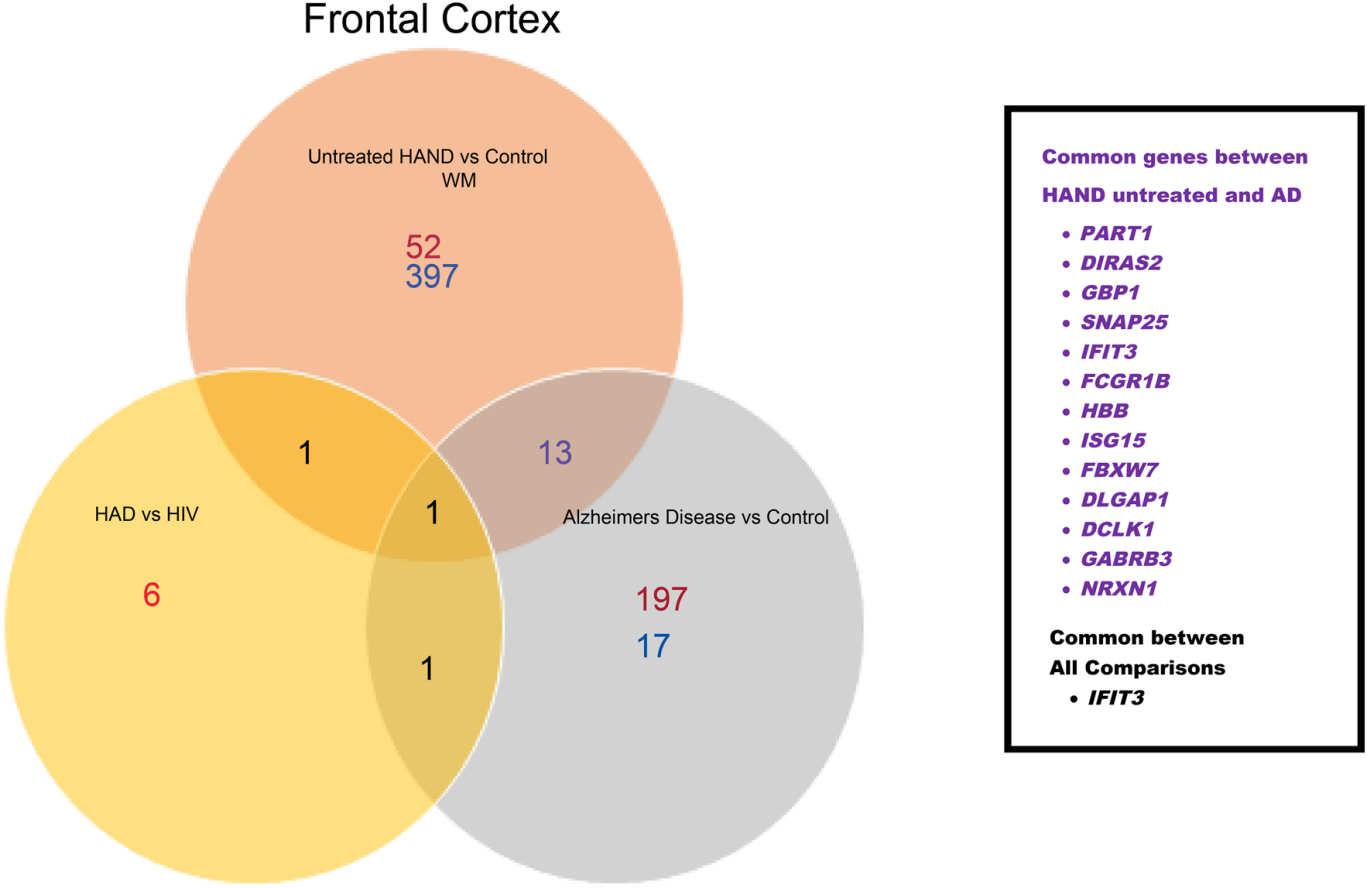
SAM results in comparison of untreated HAND and AD patients (frontal cortex). This Venn diagram represents the SAM results that were considered significant (q-value =  < .05). Both SAM analyses were done independently and then compared. The frontal cortex regions of AD patients had 17 upregulated genes (blue) and a total of 197 downregulated genes (red). The frontal cortex of HAD patients showed a total of 6 downregulated genes (red). The white matter of untreated HAND patients had a total of 397 upregulated genes (blue) and 52 downregulated genes (red). Untreated HAND patients and AD patients shared 13 common genes. However, the only common differentially expressed gene based on mRNA expression was *IFIT3*.

**Figure 10:**
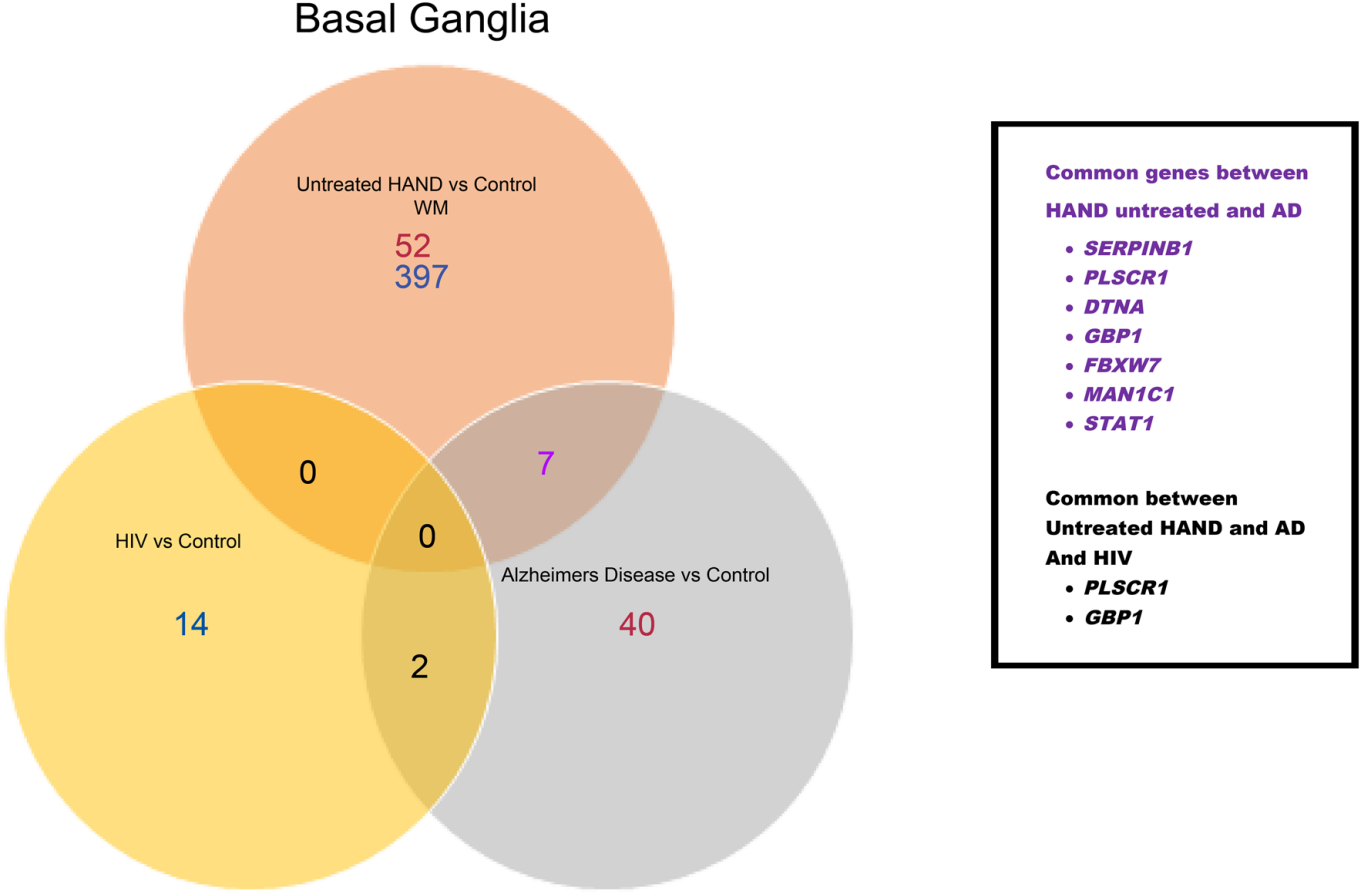
SAM results in comparison of untreated HAND and AD patients (basal ganglia). This Venn diagram represents the SAM results that were considered significant (q-value =  < .05). Both SAM analyses were done independently and then compared. The basal ganglia regions of AD patients had 40 downregulated genes (red). While the HIV-1 patients only had 14 genes up-regulated (blue) based on mRNA expression. The white matter region of untreated HAND patients had a total of 397 upregulated genes (blue) and 52 downregulated genes (red). In comparison, untreated HAND and AD shared 7 genes in common seen in purple. While HIV-1 patients only had *PLSCR1 and GBP1* in common with AD and untreated HAND patients.

**Figure 11:**
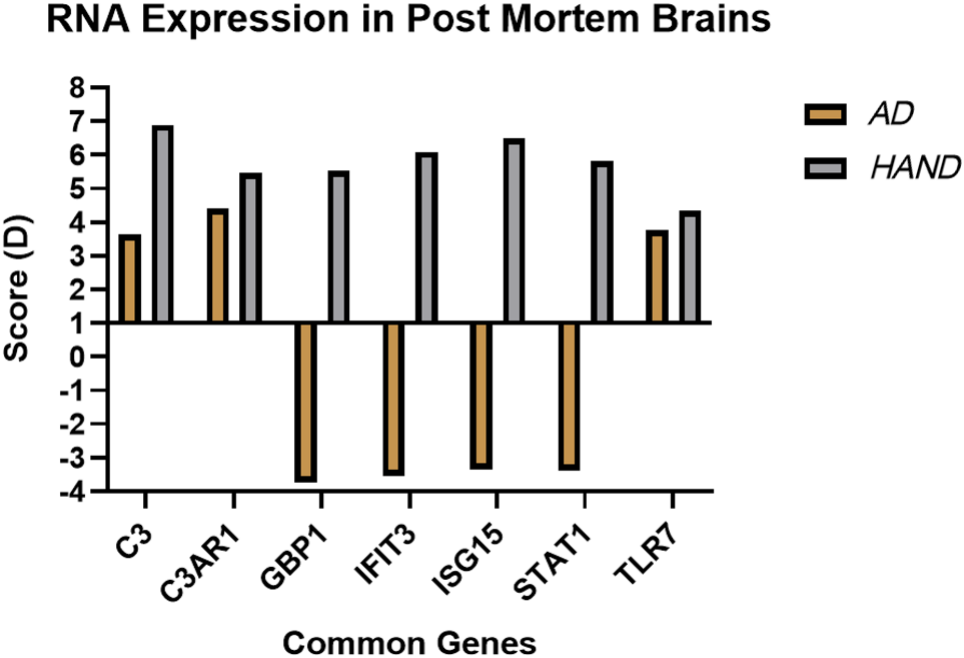
Dysregulated RNA in AD and HAND. This graph represents the dysregulation of RNA expression in HAND and AD. The results were derived from the SAM analysis. Two unpaired analysis was done independently “Control vs AD” and “Control vs HAND”. The Score (D) represented negative values which represent down-regulation of RNA and positive values represent upregulation in RNA expression. The dark yellow represents “AD” data and the grey represents “HAND”. The common genes that were derived from the SAM Analysis results and submitted through DAVID for the gene ontology analysis.

Table S2 (in the supplementary materials) contains DEGs identified through the SAM analysis. The study was focused on identifying the common DEGs in AD and untreated HAND or cART-treated HAND post-mortem brain samples by comparing them to control groups individually. Compared to the healthy control, the treated HAND patient data resulted in 25 upregulated and 19 downregulated genes. When comparing untreated HAND patients to the healthy control, 52 downregulated genes and 397 upregulated genes were identified. The AD data contained three platforms, including GPL 570, which generated 385 upregulated genes and 62 downregulated genes. Figure 8 represents a Venn diagram comparing the SAM results from untreated HAND and AD subjects. A total of 30 genes were commonly dysregulated, including *C3* (untreated HAND: *d-score*:6.894, *fold change*: 14.104, *q*: < 0.001; AD: *d-score*: 3.623, *fold change*: 1.971, q: < 0.001), *C3aR1* (untreated HAND: *d-score*: 5.468, *fold change*: 4.018, *q*: < 0.001; AD: *d-score*: 4.400, *fold change*: 1.867, *q*: < 0.001), *TLR7* (untreated HAND: *d-score*: 4.345, *fold change*: 8.197, *q*: 0.001; AD: *d-score*: 3.756, *fold change*: 1.945, *q*: 0.001), and *DTNA* (untreated HAND: *d-score*: 5.468, *fold change*: 4.018, *q*: 0.001; AD: *d-score*: 4.850, *fold change*:1.916, *q*: 0.001). The platform GPL96 contained basal ganglia regions that generated 40 downregulated genes, while the frontal lobe regions generated 109 downregulated genes and 17 upregulated genes. The AD data found in GPL97 had no DEGs in the basal ganglia regions and only 88 downregulated genes in the frontal lobe regions. The results from GPL96 and GPL97 were averaged and used to compare with HAND subject data. Due to the lack of data from other brain regions in one HAND dataset, Fig. 9 only represents the frontal cortex and white matter data. A total of 14 genes were found shared between untreated HAND and AD, including *IFIT3* (untreated HAND: *d-score*: *6.086, fold change*: 10.542, *q*: < 0.001) (AD: *d-score*: *−3.628*, *fold change*: 0.580, *q*: < 0.001), *ISG15* (untreated HAND: *d-score: 6.491, fold change*: 11.531, *q*: < 0.001, AD: *d-score*: *−3.342*, *fold change*: 0.611, q: < 0.001), and *GBP1* (untreated HAND: *d-score: 5.530, fold change*: 9.495, *q*: < 0.001, AD: *d-score*: *−3.352, fold change*:0.585, *q*: < 0.001). Nonetheless, Fig. 9 did not have any gene expression results from the white matter of AD brains as it was not available. Figure 10 represents data derived from the basal ganglia in which eight common genes, including *STAT1* and *GBP1*, were found shared between untreated HAND and AD. As illustrated in Fig. 11, these genes had an opposite expression in HAND and AD patients compared to controls.

Interestingly, a total of 15 DEGs were shared between cART-treated and untreated HAND patients with an opposite expression that depended on treatment. For example, *C4orf3* (cART untreated HAND: *d-score*:-4.6960.580 fold change, q: < 0.001, treated HAND: *d-score*: 4.832, *fold change*: 2.0203, *q*: < 0.001) was upregulated in cART treated HAND subjects but downregulated in cART untreated HAND patients. Only two genes, *STK32A* (treated HAND: *d-score*: 3.767, *fold change*:1.965, *q*: 0.001, AD: *d-score*: 3.532, *fold change*: 1.554, *q*: < 0.001) and *CD24* (cART treated HAND: *d-score*: −4.079, *fold change*: 0.0984, *q*: < 0.001; AD: *d-score*: 3.460, *fold change*: 2.081, *q*: < 0.05), were found to be common DEGs between cART treated HAND and AD respectively. For this reason, our focus was aimed at the cART untreated HAND subject data. There were many more genes in common between cART untreated HAND and AD patients' data when regions of the brains were not considered, with a total of 51 common genes found dysregulated compared to healthy controls. The independent SAM results indicated that a total of 37 genes were upregulated in cART untreated HAND. Out of these 37 genes in HAND, 27 genes were upregulated, and ten were downregulated in an AD patient's sample. As previously mentioned an additional comparison using white participant-only data was analyzed. The comparison of the basal ganglia regions between AD vs. Control in GPL96 showed significant downregulation in *ISG15* (AD: *d-score*:−4.095, *fold change*: 0.394, *q*: < 0.001) and *STAT1* (AD: *d-score*:−3.909, *fold change*: 0.510, *q*: < 0.001). In the frontal cortex regions found in GPL97 there was a downregulation in *IFIT3* (AD: *d-score*:−4.249, *fold change*: 0.577, *q*: < 0.001) and *GBP1* (AD: *d-score*:−3.788, *fold change*: 0.578, *q*: < 0.001). The frontal cortex region found in GPL96 demonstrated a downregulation in *IFIT3* (AD: *d-score*:−4.980, *fold change*: 0.609, *q*: < 0.001), *ISG15* (AD: *d-score*:−5.099, *fold change*: 0.518, *q*: < 0.001), *GBP1* (AD: *d-score*:−4.439, *fold change*: 0.573, *q*: < 0.001) and *STAT1* (AD: *d-score*:−4.247, *fold change*: 0.658, *q*: < 0.001). SAM analysis was used to identify minor changes that were significant between control and disease models. Although the target genes were not significant as observed in linear models, the false discovery rate was adjusted based on sample size using SAM for a more reliable observation^31^. These results suggested the data was not skewed by the presence of minorities (Black, Asian, Hispanic) in the original analysis.

Table S3 (in the supplementary materials) contains DEGs identified through the SAM analysis. When comparing the basal ganglia between different patients' groups' results among HAD vs. control, only 3 genes*, GUSB11* (*d-score*: 2.409, *fold change*: 13.3301, *q*: < 0.001) and, *TNFRSF1B* (*d-score*: 2.116, *fold change*: 4.630, *q*: < 0.001), and *PLA1A* (*d-score*: 1.985, *fold change*: 4.109, *q*: < 0.001), were considered significantly upregulated. In the comparison between HIV-1 and controls, 14 genes were upregulated in the HIV-1 group, including *GBP1* (*d-score*: 2.298, *fold change*: 9.113, *q*: < 0.001), *IFIT3* (*d-score*: 2.022, *fold change*: 3.870, *q*: < 0.001), and *ISG15* (*d-score*: 1.998, *fold change*: 4.786, *q*: < 0.001). The HIV-1 vs. HAD data analysis found that only two genes, *ZNF808* (*d-score*: −1.835, *fold change*: 0.1498, *q*: < 0.001) and *IFI6* (*d-score*: −1.803, *fold change*: 0.248, *q*: < 0.001), were downregulated. In the white matter between HAD vs. control, there were 4 downregulated genes: *LRRN1* (*d-score*: −2.720, *fold change*: 0.239, *q*: < 0.001), *TPBG* (*d-score*: 2.617, *fold change*: 0.377, *q*: < 0.001), *CAP2* (*d-score*: −2.580, *fold change*: 0.317, *q*: < 0.001), and *NNAT* *(d-score*: −2.439, *fold change*: 0.301, *q*: < 0.001). The *IFI4L* (*d-score*: 3.378, *fold change*: 19.433, *q*: < 0.001) was the only upregulated gene between HIV-1 vs. control. A total of 5 genes were found upregulated between HAD and HIV-1 subjects. There were no significant results within the frontal cortex of HAD vs. control subjects. The 6 genes that were downregulated between HAD and HIV-1 subjects were *BTN3A3* (*d-score*: −2.876, *fold change*: 0.307, *q*: < 0.001), *ANXA1* (*d-score*: −2.825, *fold change*: 0.429, *q*: < 0.001), *TIMP1* (*d-score*: −2.749, *fold change*: 0.184, *q*: < 0.001), *MX1* (*d-score*: −2.663, *fold change*: 0.440, *q*: < 0.001), *GADD45A* (*d-score*:−2.562, *fold change*: 0.367, *q*: < 0.001), and *IFIT3* (*d-score*: −2.4896, *fold change*: 0.259, *q*: < 0.001). Other genes that appeared in the list of downregulated genes but did not have significant q values were *STAT1* (*d-score*: −2.037, *fold change*: 0.406, *q*: 0.141)*, GBP1* (*d-score*: −2.032, *fold change*: 0.216, *q*: 0.141)*,* and *ISG15* (*d-score*: −2.271, *fold change*: 0.262, *q*: 0.128). Details of the full list of genes that were up and downregulated among different patient groups across three brain regions can be found in Table S3. Table 2 summarizes the results of the SAM analysis, specifically the genes that were common between both pathologies. Figure 11 shows a visual representation of the genes that were more frequently observed throughout comparisons along with their expression levels in AD or HAND subjects, including *ISG15, GBP1, STAT1, IFIT3,* and *TLR7.* Some of the significant DEGs genes using the linear regression model and SAM analysis are *ISG15*, *STAT1,* and *IFIT3*.

**Table 2:** Common differentially expressed genes between AD and HAND patients.

Gene	Function	Comparison and up or downregulation	Brain region
*ISG15*	Defense response to virus type I interferon signaling pathway	HAND untreated vs control-Down AD vs control FC-Up HAD vs HIV-1 BG-Up HAD vs HIV-1 FC-Down	White matter Frontal cortex Basal ganglia
*GBP1*	Defense response to virus interferon-gamma-mediated signaling pathway	AD vs control FC-Up AD vs control BG-Up HAND untreated vs control- Down HAD vs HIV-1 FC-Down	Frontal cortex Basal ganglia White matter
*IFIT3*	Defense response to virus type I interferon signaling pathway	AD vs control FC-Up HAND untreated vs control- Down HAD vs HIV-1 BG Up HAD vs HIV-1 FC Down	Frontal cortex White matter Basal ganglia
*PLSCR1*	Defense response to virus response to interferon-beta	HAND untreated vs control- Down AD vs control BG-Up	White matter Basal ganglia
*STAT1*	Defense response to virus interferon-gamma-mediated signaling pathway, Type I interferon signaling pathway response to interferon-beta	HAND untreated vs control- Down AD vs control BG-Up HAD vs HIV-1 FC-Down	White matter Basal ganglia Frontal cortex
*DLGAP1*	Chemical synaptic transmission	AD vs control FC-Up HAND untreated vs control- Up	Frontal cortex White matter
*FCGR1B*	Interferon-gamma-mediated signaling pathway	HAND untreated vs control- Down AD vs control FC-Up	White matter Frontal cortex
*DTNA*	Chemical synaptic transmission, Signal transduction	HAND untreated vs control- Down AD vs control BG-Up Nucleusaccumbens/amygdala-Up	White matter Basal ganlgia Nucleus accumbens /amygdala
*NRXN1*	Chemical synaptic transmission, Signal transduction	HAND untreated vs control- Up AD vs control FC-Up	White matter Frontal cortex
*SNAP25*	Chemical synaptic transmission	HAND untreated vs control- Up AD vs control FC-Up	Frontal cortex White matter
*ANXA1*	Signal transduction, Inflammatory response	HAND untreated vs control- Down Nucleusaccumbens/amygdala-Down	White matter Nucleus/amygdala
*DDR2*	Signal transduction, Regulation of bone mineralization	HAND untreated vs control- Down Nucleusaccumbens/amygdala-Down	White matter Nucleus/amygdala
*GABRB3*	Signal transduction	HAND untreated vs control- Up AD vs control FC-Up	White matter Frontal cortex
*ITPR2*	Signal transduction	HAND untreated vs control- Down Nucleus accumbens/amygdala-Down	White matter Nucleus accumbens/amygdala
*BMP2K*	Regulation of bone mineralization	HAND untreated vs control- Down Nucleus accumbens/amygdala-Down	White matter Nucleus accumbens /amygdala
*C3*	Positive regulation of vascular endothelial growth production, inflammatory response Regulation of complement activation	HAND untreated vs control- Down Nucleus accumbens/amygdala-Down	White matter Nucleus accumbens/amygdala
*C3AR1*	Positive regulation of vascular endothelial growth production inflammatory response Regulation of complement activation	HAND untreated vs control- Down Nucleus accumbens/amygdala-Down	White matter Nucleus accumbens/amygdala
*TLR7*	Inflammatory response	HAND untreated vs control- Down Nucleus accumbens/amygdala-Down	White matter Nucleus accumbens/amygdala

This table summarizes the results from SAM analysis and Gene Ontology Analysis. The genes and the processes they are involved in are listed. The comparison column includes the comparison (HAND or AD) used for SAM analysis and whether they were up or downregulated. The specific region is represented by abbreviations (FC: frontal cortex, BG: basal ganglia, WM: white matter). Four mRNA transcripts were significantly differentially expressed between comparisons they are *ISG15, GBP1, STAT1, and IFIT3.*

### Processes of common genes *ISG15, GBP1, IFIT3, PLSCR1, STAT1, TLR7* involved in both AD and HAND

**Figure 12:**
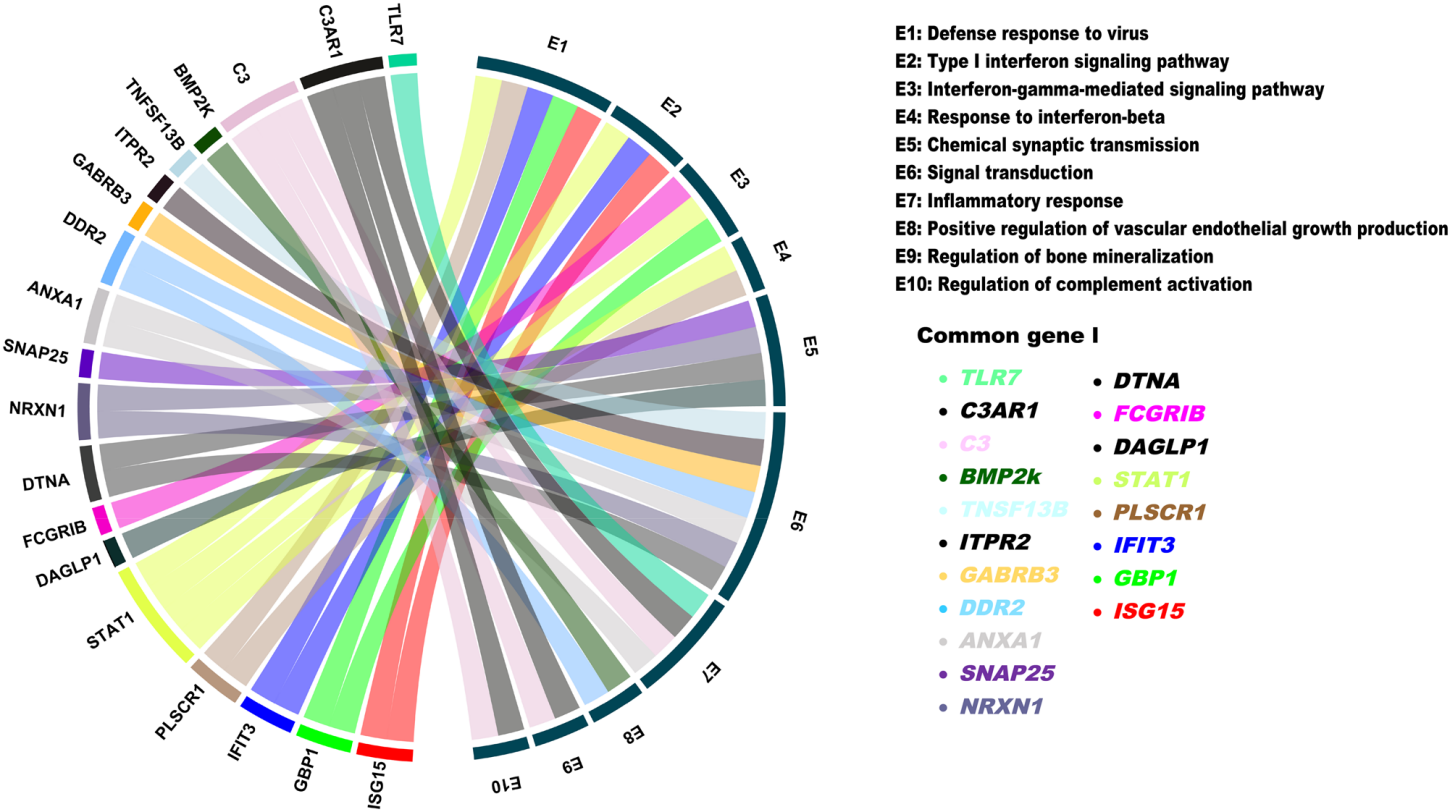
Chord diagram of mRNA and respective functions. This chord diagram represents the common genes that were derived from the SAM Analysis results and submitted through DAVID for the Gene ontology analysis. Each “chord” connects a gene to its respective function or process. In this graph, genes may be involved in multiple processes.

The 51 common genes between AD and untreated HAND were submitted to DAVID. As a result, our list majorly contributed to a total of 10 processes. Figure 12 represents the following genes and their respective functions, including the defense response to the virus, interferon-gamma-mediated signaling pathway, type 1 interferon signaling pathway, response to interferon-beta, chemical synaptic transmission, signal transduction, positive regulation of vascular endothelial growth factor, inflammatory response, and regulation of complement activation. Out of the 51 genes, 6 of them, *ISG15, GBP1, IFIT3, PLSCR1, STAT1,* and *TLR7*, were part of the defense response of the virus. A total of 8 genes are involved in signal transduction, including *ANXA1, C3, DDR2, DTNA, GABRB3, ITRP2, NRXN1,* and *TNFSF13B*. There were three genes involved in the type I interferon signaling pathway, *ISG15, IFIT3,* and *STAT1*. From the list, *STAT1* and *PLSCR1* genes are involved in response to interferon beta. Both *C3* and *C3AR1* are involved in regulating complement activation and vascular endothelial growth production. *DLGAP1, DTNA*, *NRXN1,* and *SNAP25* genes are involved in chemical synaptic transmission. The inflammatory response genes from our list included *ANXA1, C3, C3AR,* and *TLR7*. The final process that our gene list was involved in was the regulation of bone mineralization which includes *BMP2K* and *DDR2*. The more frequently involved genes were *STAT1, C3,* and *C3AR1.*

### Pathway Analysis to identify key pathways involving *CD44*, *FCGR1B*, *GBP1*, and *STAT1* commonly found in AD and HAND datasets

The list of common genes was submitted to DAVID, which generated 3 different REACTOME pathways. One of the REACTOME pathways was the interferon-gamma signaling pathway, which contained the genes *CD44, FCGR1B, GBP1,* and *STAT1*. The interferon-alpha/beta signaling pathway was the second pathway, which contained *ISG15, IFIT3,* and *STAT1*. The final pathway was the regulation of the complement cascade which included *C3* and *C3AR1*. More details on genes and the processes are listed in Table 2.

### Discussion

The neurodegeneration observed in populations suffering from HAND and AD share common characteristics, including neuroinflammation and amyloid-beta deposition in the CNS^3,33^. Considering these neuropathologies are relatively common among the aging population with HIV-1 infection, it has become increasingly difficult to detect and distinguish between these two diseases based on their symptoms^2,3,5^. To biologically characterize HAND and AD and find a potential biological indicator^1^, it is important to understand the underlying molecular pathways involved in these two diseases, especially in co-existing conditions^6^. Therefore, the present study has taken advantage of the existing RNA expression datasets from post-mortem brain samples to investigate a set of common and distinctive gene biomarkers for HAND and/or AD. The AD, untreated HAND, and cART-treated HAND data were initially compared to healthy controls considering that AD and HAND will have similar gene expression compared to the control. Using independent results with this same analysis, we were able to perform this type of comparison. Due to the relatively small number of common genes between AD and cART-treated HAND patient transcriptome data, the treatment was not considered in our analysis. Our objective was to identify common mRNA transcripts differentially expressed between HAND and AD patients’ brains irrespective of their age. The basal ganglia and the frontal cortex regions of the AD patients' post-mortem brains were investigated in this study. Even though the AD dataset has a much larger number of study participants than the HIV-1 datasets, the current study helped to identify dysregulated signaling pathways that are crucial in developing the neuropathology of AD and/or HAND.

Type 1 interferons are part of the innate immune response that interferes with viral infection. Toll-like receptors allow cells to recognize bacteria, viruses, and cellular debris and activate interferons' transcription. The current study has shown an upregulation of *TLR7* in untreated HAND and AD post-mortem samples. TLR7 is involved in the recognition of viruses, double-stranded RNA, and single-stranded RNA, and its activation results in downstream transcription of type 1 interferons, including interferon α/ß (IFN-α/ß)^34^. Interaction of IFN-α/ß and its receptor produces the IFNAR complex, which recruits *STAT1* and *STAT2* for phosphorylation and creates a heterodimer that forms a complex with IRF9, known as ISGF3^35^. This complex is then translocated into the nucleus, activating the transcription of interferon-stimulated genes (ISGs) as part of the immune response^36^. This interferon response is known as the JAK-STAT pathway signaling cascade that is known to promote neuroinflammation. Therefore, it is important to investigate how to regulate the immune response to avoid dysregulated forms of inflammation in the brain that are commonly observed in neurodegenerative diseases^37^. The results from the DAVID analysis allowed the identification of two common interferon pathways that were found dysregulated in both AD and HAND (Fig. 12). Three genes were identified to be involved in the type I interferon pathway, including *STAT1, IFIT3*, and *ISG15*. Several studies with genomic and transcriptome analysis have indicated that genes like *ISG15*, *STAT1*, and *IFIT3* can, directly and indirectly, regulate HAND or AD^38–43^. However, their underlying mechanisms and contribution to neuropathology are not well understood. Our study has established that in the white matter of untreated HAND subjects, the expression of *STAT1*, *IFIT3*, and *ISG15* was upregulated as expected due to viral infection. However, in AD post-mortem brain samples, there was a downregulation of *STAT1* in the basal ganglia and a downregulation of *ISG15*, and *IFIT3* in the frontal cortex. This is supported by another study analyzing the post-mortem AD brain, where decreased *IFN-α* and *IRF7* levels suggest an impaired immune response. The study also demonstrated that the expression of genes varies in distinct regions of the brain, which may have different consequences due to region- and gene-specific functions^44^. In our analysis, the frontal cortex and basal ganglia regions were analyzed based on their involvement in motor commands and executive function, respectively.

The type 1 interferon response can have harmful or beneficial effects that have been seen in various neurodegenerative diseases. Roy et al. have established that *STAT1*, or a downstream component of the JAK-STAT pathway, protects against IFN-α-mediated injury in the CNS^45^. Previous studies have aimed to understand the function of type I interferon in the CNS and to confirm the upregulation of interferons in the presence of amyloid-beta due to chronic stimulation^37,46^. However, our results showed a downregulation of *STAT1* in AD post-mortem brains. This observation was supported by another study where the initial upregulation of interferon response in microglia was downregulated within six days of infection with a neurotropic virus, indicating impaired IFN-γ response^47^. The present study has identified four genes, *CD44, FCGR1B, GBP1,* and *STAT1*, from the common genes of AD and HAND that are involved in the IFN-γ mediated signaling pathway. Previous studies have investigated patients' nitric oxide (NO) levels in different stages of AD in both microglia and astrocytes, which have neurotoxic effects in the brain and contribute to AD pathology^48^. The study also identified that IFN-γ and tumor necrosis factors (TNF) were needed to induce NO production. However, the mechanism of how these cytokines regulate NO production and how this can potentially reduce the inflammation observed in AD pathology needs further investigation^48^. Another study has suggested that experimental autoimmune encephalomyelitis (EAE) induced IFN-γ signaling that can have a protective effect through *STAT1* in immune cells of the CNS^49^. In the absence of *STAT1*, cells suffered from a severe form of EAE. *STAT1* deficiency observed in our analysis may have detrimental effects as seen in EAE CNS in vitro models^49^. However, IFN-γ produced by CD8+ T cells and microglia have been observed in the brains of mice and patients with AD^50^. The type II interferon pathway requires a *STAT1* homodimer that translocates into the nucleus. Here, it can bind to cis-regulatory elements, known as gamma activating sequences (GAS) elements, which can either activate or suppress interferon-regulated genes^51^. The study observed that phosphorylated STAT1 protein allows tau fragments (1–368) to bind and activate the transcription of *BACE1*. It was observed that this mechanism is dependent on the presence of the amyloid precursor protein (586–695), which regulates the phosphorylation of kinases, SGK1 and JAK2, which are both activators of STAT1^52^.

Some of the downstream ISGs that deserve further investigation regarding their roles in the CNS include *IFIT3* and *ISG60,* which are expressed downstream of the JAK-STAT pathway. It has been observed that interferon-beta (IFN-β) secretion activates the production of *IFIT3*, which can then regulate the downstream production of chemokine *CXCL10*^53^. *IFIT3* can function as an inhibitor of cellular and viral processes, cell migration, proliferation, signaling, and viral replication^54^. *ISG15* has been identified as an interferon-stimulated gene since its expression is induced in response to type I interferons or lipopolysaccharide treatment^41,55^. *ISG15* activity is tightly regulated by specific signaling pathways that play an innate immunity role. A study demonstrated that *ISG15* could negatively regulate the type 1 interferon response through *USP18* stabilization^41^. The results of this study showed an increase of *ISG15* in cART untreated HAND brain samples. This finding is supported by a previous study that analyzed cerebrospinal fluid (CSF) and post-mortem brain samples of people with HAND, identifying increased levels of *ISG15* in the early stages of HAND^56^. This also suggests that the regulation of downstream activation of ISGs in response to type 1 interferons may benefit people with HIV-1. Since both of these ISGs are activated downstream of STAT1 in the type 1 interferon response, understanding how these genes may regulate inflammation or other factors contributing to the neurodegeneration seen in the CNS may be helpful for the development of therapeutic drugs.

A recent study has indicated that the interferon response can directly or indirectly regulate other mechanisms involved in neurodegeneration on *C3* and *C3AR1*^46^. These two genes are involved in the complement cascade pathway and were both found upregulated in AD and HAND subjects. However, further investigation is needed to understand its contribution to neurodegeneration^46^. Innate immunity can be regulated through the complement pathway in which the cleavage of C3 into C3a and C3b is followed by binding to C3AR1 and CR3, respectively^57^. It is expressed in the CNS and is associated with aging and neuropathology^57^. The C3-C3AR signaling has been observed to mediate mechanisms involved in immune response and neuron interactions involved in network function and amyloid-beta pathology^57^. However, the interactional relationships between neurons, astrocytes, and microglia need to be further understood. This study showed that C3AR has a critical role in mediating CNS immune hemostasis and tau pathology through the JAK-STAT pathway^57^. In addition, levels of IL-6 have been observed in the brains of AD patients. The presence of amyloid-beta can activate the NALP3 inflammasome that will cause the secretion of IL-1beta and IL-6, respectively, and induce the transcription of acute-phase response proteins like *C3*^58^. The *C3* gene expression has been observed in the brains of PWH with HAND, where NF-kB induction of IL-6 activates the *C3* gene. This contributes to neuroinflammation and cognitive dysfunction in people with HIV-1 infection^59^. Further research is needed to investigate and compare the mechanisms involved in regulating *C3* and *C3AR1* in the CNS of AD and HAND subjects.

Our data support multiple studies that recognize type 1 and type II interferon signaling pathways related to HAND and AD despite the small sample size. In summary, it is important to understand further the mechanism of these pathways and how they mediate neuronal plasticity through astrocyte and microglia activation. *STAT1* is a gene involved in interferon response; however, more research is needed to understand its regulation in AD pathology. Following the activation of STAT1, genes *ISG15* and *IFIT3* are activated and are involved in innate immunity. The expression levels of these mRNA transcripts at different regions of the brain deserve further investigation to understand chronic CNS inflammation related to HAND or AD. Due to the limited available data, treatment was not considered important in HAND data analysis. However, since most people with HIV-1 receive the treatment, it would be ideal for collecting data from larger cohorts receiving treatment. In addition, there was a large mean age difference between the AD and HAND datasets which is one of the limitations as both the disease pathologies are associated with age. However, the current study was focused on the comparative gene expression analysis of AD and HAND brains as it was observed in deceased patients irrespective of their age. Another limitation would be the inconsistent sample size found in the datasets and the inconsistent brain regions analyzed. Future research should consider using an equal number of participants from diverse backgrounds as well as specific brain regions. However, as mentioned before, our results were not compromised by the presence of minorities (Black, Asian, Hispanic) in the analyzed datasets.

In conclusion, the transcriptome profiles of the brains of people with HAND and AD highlight opposite expressions of RNA involved in type 1 interferon signaling, including *STAT1, ISG15*, and *IFIT3*. The expression of genes involved in this immune response varies in different neurodegenerative diseases and is suggested to be involved in the different neuropathologies that lead to cognitive decline. Further research is needed to confirm these observations using in vitro or in vivo models to understand further the mechanisms involved in the progression of neurodegenerative diseases. Nonetheless, to the best of our knowledge, this is the first comprehensive report comparing the mRNA expression in AD and HAND from two established RNA datasets. This observation may lead to further investigation on AD and HAND pathology among older PWH for potential therapeutic strategies.

## Supplementary Information

Supplementary Information 1.

Supplementary Information 2.

Supplementary Information 3.

Supplementary Information 4.

## Data Availability

The data generated throughout the present study have been deposited to NCBI's Gene Expression Omnibus (GEO) under the GEO accession number GSE35864, GSE84422 and GSE28160.

## Abbreviations

AD: Alzheimer's disease
HIV-1: Human immunodeficiency virus 1
HAND: HIV Associated neurocognitive disorders
cART: Combination antiretroviral treatment
DE: Differentially expressed
SAM: Significance analysis of microarrays
HAD: HIV-associated dementia
HIVE: HIV encephalitis
GEO: Gene expression omnibus
ISGs: Interferon stimulated genes
EAE: Experimental autoimmune encephalomyelitis
DEGs: Differentially expressed genes
PWH: People with HIV
ANI: Asymptomatic Cognitive Impairment
