# Supplementary material for: Differential expression of interferon-induced protein with tetratricopeptide repeats 3 (IFIT3) in Alzheimer's disease and HIV-1 associated neurocognitive disorders
Source: Sci Rep. 2023 Feb 25;13:3276. doi: 10.1038/s41598-022-27276-7 (PMC9968324; doi:10.1038/s41598-022-27276-7)
Supplement: Supplementary file 1 — Supplementary Information 1. [file 41598_2022_27276_MOESM1_ESM.docx]

**Supplemental Table-S1:** Liner model comparisons for six subject groups (HAD vs control, cognitively normal vs HIV-1 vs control, HAD vs cognitively normal vs HIV-1, HAND (cART untreated) vs control, cART treated normal HIV-1 vs control, AD vs control) of all the three brain sectors.

**Supplemental Table-S2:** Results of the SAM analysis for GSE28160 and GSE84422

**Supplemental Table-S3:** SAM analysis results for GSE35864
